# Patterns and determinants of breastfeeding and complementary feeding practices in urban informal settlements, Nairobi Kenya

**DOI:** 10.1186/1471-2458-11-396

**Published:** 2011-05-26

**Authors:** Elizabeth W Kimani-Murage, Nyovani J Madise, Jean-Christophe Fotso, Catherine Kyobutungi, Martin K Mutua, Tabither M Gitau, Nelly Yatich

**Affiliations:** 1 African Population and Health Research Center (APHRC), Nairobi, Kenya; 2 University of Southampton, Centre for Global Health, Population, Poverty, and Policy, Faculty of Social and Human Science, Southampton, UK; 3 University of the Witwatersrand, MRC Mineral Metabolism Research Unit, Department of Paediatrics, Faculty of Health Sciences, Johannesburg, South Africa

## Abstract

**Background:**

The World Health Organisation (WHO) recommends exclusive breastfeeding during the first six months of life for optimal growth, development and health. Breastfeeding should continue up to two years or more and nutritionally adequate, safe, and appropriately-fed complementary foods should be introduced at the age of six months to meet the evolving needs of the growing infant. Little evidence exists on breastfeeding and infant feeding practices in urban slums in sub-Saharan Africa. Our aim was to assess breastfeeding and infant feeding practices in Nairobi slums with reference to WHO recommendations.

**Methods:**

Data from a longitudinal study conducted in two Nairobi slums are used. The study used information on the first year of life of 4299 children born between September 2006 and January 2010. All women who gave birth during this period were interviewed on breastfeeding and complementary feeding practices at recruitment and this information was updated twice, at four-monthly intervals. Cox proportional hazard analysis was used to determine factors associated with cessation of breastfeeding in infancy and early introduction of complementary foods.

**Results:**

There was universal breastfeeding with almost all children (99%) having ever been breastfed. However, more than a third (37%) were not breastfed in the first hour following delivery, and 40% were given something to drink other than the mothers' breast milk within 3 days after delivery. About 85% of infants were still breastfeeding by the end of the 11^th ^month. Exclusive breastfeeding for the first six months was rare as only about 2% of infants were exclusively breastfed for six months. Factors associated with sub-optimal infant breastfeeding and feeding practices in these settings include child's sex; perceived size at birth; mother's marital status, ethnicity; education level; family planning (pregnancy desirability); health seeking behaviour (place of delivery) and; neighbourhood (slum of residence).

**Conclusions:**

The study indicates poor adherence to WHO recommendations for breastfeeding and infant feeding practices. Interventions and further research should pay attention to factors such as cultural practices, access to and utilization of health care facilities, child feeding education, and family planning.

## Background

The first two years of life are critical stages for a child's growth and development. Any damage caused by nutritional deficiencies during this period could lead to impaired cognitive development, compromised educational achievement and low economic productivity [1-3]. Poor breastfeeding and complementary feeding practices, together with high rates of morbidity from infectious diseases are the prime proximate causes of malnutrition in the first two years of life. Breastfeeding confers both short-term and long-term benefits to the child. It reduces infections and mortality among infants, improves mental and motor development, and protects against obesity and metabolic diseases later in the life course [3-7].

The WHO recommends exclusive breastfeeding in the first six months, beginning from the first hour of life, to meet the infant's nutritional requirements and achieve optimal growth, development and health. The mother is advised to continue breastfeeding up to two years of age or more and begin nutritionally adequate, safe, and appropriately-fed complementary foods at the age of six months in order to meet the evolving needs of the growing infant [6]. The WHO/UNICEF global strategy on infant and young child feeding practices aims to promote optimal breastfeeding and complementary feeding practices, through various initiatives for example the Baby Friendly Hospital Initiative (BFHI) and the International Breastfeeding Code [8]. Interventions promoting optimal breastfeeding could prevent 13%, while those promoting optimal complementary feeding could prevent another 6% of deaths in countries with high mortality rates [5].

Poor breastfeeding and complementary feeding practices have been widely documented in the developing countries. Only about 39% of infants in the developing countries, 25% in Africa are exclusively breastfed for the first six months. Additionally, 6% of infants in developing countries are never breastfed [9]. In Kenya, according to Kenya Demographic and Health Survey 2008-2009 [10], 32% of children under the age of six months are exclusively breastfed, improving from only 13% in 2003 [11]. As a result, substantial levels of child malnutrition and poor child health and survival have been documented in Kenya [11]. Deriving from the broad principles of the joint WHO and UNICEF's Global Strategy for infant and young child feeding developed in 2002 [8], the government of Kenya is implementing a strategy aimed at improving infant and young child feeding practices in Kenya. The strategy is actualized through revitalization of the BFHI [12].

Urban poor settlements or slums present unique challenges with regards to child health and survival. Slums in sub-Saharan Africa are expanding at a fast rate, and the majority of urban residents now live in slum settlements, [13]. These slums are characterized by poor environmental sanitation and livelihood conditions [14-16]. Contrary to the long-held belief that urban residents are advantaged with regards to health outcomes, urban slum dwellers tend to have very poor health indicators [14,17,18]. For example, in Kenya, slum children are reported to be sicker and to have higher mortality rates than any other sub-group in Kenya including the rural areas [14]. In line with this, infants born to mothers that reside in the urban slums may be exposed to sub-optimal breastfeeding and complementary feeding practices.

Various factors associated with sub-optimal breastfeeding and complementary feeding practices have been identified in various settings. These include maternal characteristics such as age, marital status, occupation, and education level; antenatal and maternity health care; health education and media exposure; socio-economic status and area of residence; and the child's characteristics including birth weight, method of delivery, birth order, and the use of pacifiers [19-21]. However, there are conflicting findings with regards to the consistency of the associations and the magnitude of the effects [20,22-25], suggesting that the context may be important when trying to isolate characteristics and practices that may be amenable to interventions. There is limited evidence on breastfeeding and infant feeding practices in urban slums in sub-Saharan Africa since few studies have focused on urban slums. Although there is national level evidence on breastfeeding and infant feeding practices, the data for urban areas is not disaggregated, and hence, the dearth of evidence on practices in urban slums. In this study, we have collected longitudinal data and assessed infant feeding practices with reference to WHO recommendations, in two slums in Nairobi, Kenya, and their determinants.

## Methodology

### Study setting and data source

The study was carried out in two urban slums of Nairobi Kenya (Korogocho and Viwandani) where the African Population and Health Research Center (APHRC) runs a health and demographic surveillance system; the Nairobi Urban Health and Demographic Surveillance System (NUHDSS). The two slum areas are densely populated (63,318 and 52,583 inhabitants per square km, respectively), and are characterized by poor housing, lack of basic infrastructure, violence, insecurity, high unemployment rates, and poor health indicators [14,15,17]. The socio-economic status of the two slums differs slightly: Viwandani has relatively higher levels of education and employment as being located in the industrial area, it attracts migrant workers. On the other hand, the population of Korogocho is more stable since on average, residents in the area have lived the area for a longer period than Viwandani residents. Approximately two thirds of married men live with their spouses in Korogocho, compared with half in Viwandani.

The NUHDSS involves a systematic recording (every four months) of vital demographic events including births, deaths and migrations occurring among residents of all households in the NUHDSS area, since 2003. Other data that are collected regularly include household assets, morbidity, and highest educational attainment. This paper is based on data from a maternal and child health component of a broader longitudinal study entitled "Urbanization, Poverty and Health Dynamics in sub-Saharan Africa" that was nested within the NUHDSS. The study started in February 2007 and ended in December 2010. All women who gave birth since September 2006 and their children were enrolled in the study and were followed up every four months to obtain data including self-reported health status, breastfeeding, complementary feeding practices, vaccination and health care. Data on socio-economic status was extracted from the NUHDSS database and linked to the study participants through their household identifier.

#### Data collection & sample

The data presented in this paper were collected at baseline and during the first two updates, four months apart, for each child recruited between February 2007 and May 2009. Therefore, the follow-up period for each child was on average nine months and the average age of the children at the second update was 15 months. However, we restrict our analyses to the first year of life for all 4,299 children who were enrolled in the study. Table 1 presents the sample size for the children involved in the study and the data collection dates. Six cohorts of children enrolled during the study period are included.

**Table 1:** Sample Size, Nairobi informal settlements, Kenya

	Survey 1	Survey 2	Survey 3	Survey 4	Survey 5	Survey 6	Survey 7	Survey 8	Number of observation
**Survey period ==>**	**Feb-Apr 2007**	**Jul-Aug 2007**	**Oct-Dec 2007&Mar-Apr 2008**	**May-Aug 2008**	**Sep 2008-Jan 2009**	**Feb-May 2009**	**Jun-Sep 2009**	**Oct-Jan 2010**	**Feb 2007-Sept 2009**

Panel 1	**615**	507	378	*	*	*	*	*	1,500
Panel 2		**458**	359	323	*	*	*	*	1,140
Panel 3			**948**	727	645	*	*	*	2,320
Panel 4				**968**	814	696	*	*	2,478
Panel 5					**479**	398	333	*	1,210
Panel 6						**831**	689	498	2,018
**Total**	**615**	**965**	**1,685**	**2,018**	**1,938**	**1,925**	**1,022**	**498**	**10,666**

* Censored

Note: The total number of children enrolled across all six panels is 4299

#### Variables

##### a. Dependent variables

The two dependent variables are cessation of breastfeeding and introduction of complementary foods (liquids and solids). Cessation of breastfeeding is a time-dependent variable indicating the age when breastfeeding was stopped. Introduction of complementary foods is also a time-dependent variable indicating the age at which complementary foods (either liquids or solids other than breastmilk) were introduced. Children who entered the study after the events (breastfeeding cessation or initiation of complementary foods) had occurred had their information updated retrospectively. About 1% of children were never breastfed and were excluded from survival analysis. Children who did not have their information updated due to loss to follow-up were excluded from the study.

##### b. Independent variables

The independent variables were: the child's sex; the mother's age (< 25 years, 25-34 years, 35+ years); the marital status of the mother (in union i.e. currently married or living with someone; previously in union; and never married/in union); the ethnicity of the mother (Kikuyu, Luhya, Luo, Kamba, and other tribes); the highest level of education (none, primary, secondary or higher); and parity (1, 2, 3+); pregnancy desirability of the index child (wanted at the time of conception, wanted later and never wanted,); the place of delivery (health facility, home or traditional birth attendant [TBA]); the mother's perception of the child's size at birth (normal, smaller than normal, larger than normal); the socio-economic status of the household; and the slum of residence (Viwandani or Korogocho). The household socioeconomic status was defined using the household monthly expenditure per capita, taking a child to be the equivalent of half an adult. The expenditure data used was obtained from the poverty component of the Urbanization, Poverty and Health Dynamics study, and was collected in the same year as the data for the dependent variables above. This variable was recoded as tertiles of "poorest", "middle" and "least poor".

#### Data Analysis

The survival analysis of the duration of breastfeeding and the time to introduction of complementary foods is presented using Kaplan-Meier survival curves. Cox regression analysis was performed to determine factors associated with breastfeeding cessation during infancy and early introduction of complementary foods. Some independent variables had missing values, mainly maternal level variables such as the mother's age, ethnicity, the highest level of education; and the household's socio-economic status. A missing category was created in the multivariate analysis to keep all cases in the analysis (though results for missing category are not shown). In all cases, except the household expenditure variable, the number of cases with missing information was less than 10%. About one-third of the participants were missing data on household expenditure, therefore imputation using a linear interpolation computation procedure was used. A p-value of less than 0.05 was used as the cut-off for statistical significance.

### Ethical Considerations

The Urbanization, Poverty and Health Dynamics study was approved by the Ethical Review Board of the Kenya Medical Research Institute (KEMRI). The field workers were trained in research ethics and obtained informed consent from all respondents. The NUHDSS has also been approved by KEMRI's Ethical Review Board. Verbal consent is routinely obtained from all the NUHDSS respondents.

## Results

### Sample Characteristics

Almost all the children (99%) were ever breastfed; however, more than a third (37%) were not breastfed in the first hour following delivery. The main reasons given for not initiating breastfeeding immediately were: little or no breastmilk (35%); baby being asleep/tired (23%); baby being sick (13%); and mother being sick (9%). Two in five of the children were given something to drink other than the mothers' breast milk within 3 days following delivery. The main reasons given were that the mother had little or no breast milk (42%) or that the child had an upset stomach (32%). During each data collection round, mothers and other caregivers were asked if the child was being given any liquid or solid food to complement breastfeeding, with the recall period being the last three days. The most common complementary foods being given to children before the age of six months were: plain water (56%), with most children on it having been given within the first month (69%); porridge (54%), with most children on it having been introduced between the second and third months (64.7%); fresh or powdered milk (45%) with most children on it also having been introduced between second and third months of life (57%); and sweetened/flavoured water (41%), with most of children on it having been introduced within the first month of life (78%). The main reason given for introducing complementary foods to children below six months was that the mother had no or little breast milk (approx 40%). About two-thirds knew that complementary feeding should be started at 6 months while about 30% indicated they should be started before six months.

### Survival Analysis

#### Cessation of breastfeeding and introduction of complementary foods

**Figure 1:**
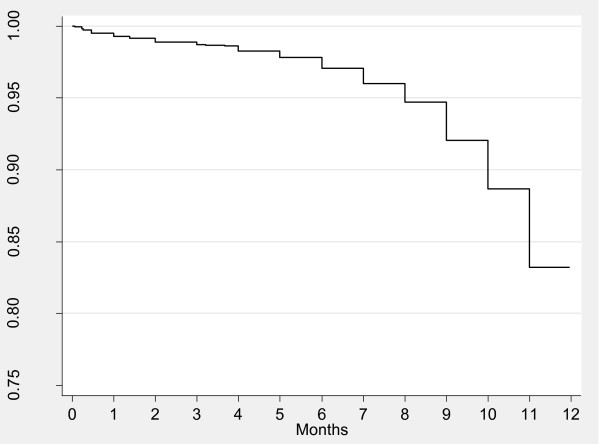
**Probability of continuing breastfeeding for specific ages, Nairobi informal settlements, Kenya**.

**Figure 2:**
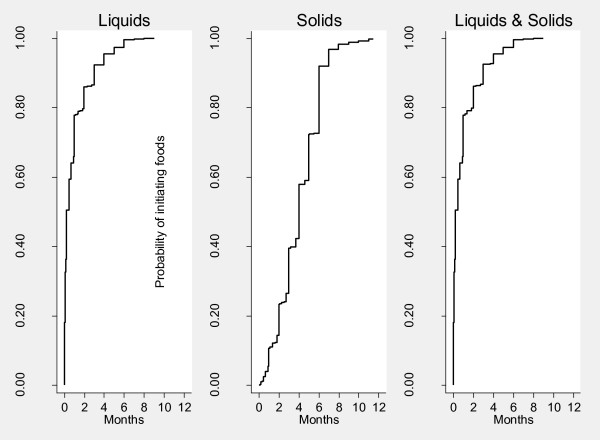
**Probability of initiating complementary foods for specific ages, Nairobi informal settlements, Kenya**.

Figure 1 shows Kaplan-Meier survival curves for probability of continuing breastfeeding at specific ages. Nearly all of the infants were started on breastfeeding, and about 85% of the infants were still breastfeeding by the end of the 11th month. Figure 2 illustrates Kaplan-Meier survival curves for time to introduction of complementary foods. Nearly all (98%) of children had been introduced to complementary foods (either liquids or solids) by the age of 6 months. Liquids were introduced much earlier than solids; the mean age of introduction of liquids was one month while that for solids was three and a half months.

#### Proportional Hazards Regression

In the multivariate analysis, the factors significantly associated with cessation of breastfeeding during infancy were the mother's marital status, her ethnicity, and her highest level of education and the perceived size of the child at birth (p < 0.05, respectively). The mother's age was marginally associated (p > 0.05 < 0.1) with the duration of breastfeeding. When other factors were controlled for, mothers who were previously in union and those never in union had close to 3 and 2 fold higher hazards of stopping breastfeeding before the age of 12 months respectively compared to mothers in union. Luhya, Luo and mothers of other ethnic groups were more likely to stop breastfeeding early (Hazard Ratios: 1.6, 1.8. and 2.1, respectively), compared to Kikuyu mothers; mothers with secondary or tertiary education level had 40% lower hazards of stopping breastfeeding compared to those with less than secondary level education; and children who were perceived as larger than normal were 40% less likely to stop breastfeeding before the age of 12 months (Table 2).

**Table 2:** Characteristics of study participants, Nairobi informal settlements, Kenya

Category	(%)
Sex	
Girls	50.5
Boys	49.6
Mother's age	
< 25 years	53.6
25-34 years	32.5
35+ years	6.4
Missing	7.5
Mother's Marital Status	
In union currently	83.3
Ever in union	7.2
Never in union	9.2
Missing	0.3
Mother's ethnicity	
Kikuyu	24.1
Luhya	16.0
Luo	18.0
Kamba	18.8
Other	15.7
Missing	7.5
Mother's education level	
< Secondary	70.7
Secondary+	21.9
Missing	7.5
Mother's Parity	
One (ref)	33.0
Two	27.8
Three+	39.0
Missing	0.3
Pregnancy desirability*	
Wanted at conception	49.8
Wanted later	37.2
Never wanted	12.4
Missing	0.5
Delivery place*	
Health Facility	71.4
Home	28.3
Missing	0.3
Perceived size at birth*	
Normal	69.5
Smaller	14.2
Larger	15.6
Missing	0.7
SES (Expenditure) Category	
Poorest	55.0
Middle	29.0
Least Poor	7.3
Missing	8.6
Slum of residence	
Korogocho	52
Viwandani	48

*Refers to the index child

Factors associated with early introduction of complementary foods (before the age of six months) at multivariate level include child's sex; mother's marital status, ethnicity, and education level; pregnancy desirability; place of delivery; and slum of residence (p < 0.05, respectively). Boys were more likely to be introduced to foods early; mothers never in union had 23% higher hazards of introducing complementary foods before 6 months of age; Luos, Luhyas and other ethnic groups were more likely to introduce foods early; mothers with at least secondary level education had 10% lower hazards of introducing foods early; children who were not wanted at conception but wanted later had 7% lower hazards of being introduced early; and mothers in Viwandani had 10% lower hazards of introducing complementary foods early compared to mothers in Korogocho (Table 3).

**Table 3:** Cox regression for cessation of breastfeeding and introduction of complementary foods, Nairobi informal settlements, Kenya

Variable	Cessation of Breastfeeding				Initiation of Complementary Foods			
	**Univariate**		**Multivariate**		**Univariate**		**Multivariate**	

	**HR**^**1**^	**95%CI**	**HR**	**95%CI**	**HR**	**95%CI**	**HR**	**95%CI**

Sex								
Girls	Ref		Ref		Ref		Ref	
Boys	0.98	[0.80, 1.21]	0.88	[0.71, 1.10]	1.03	[0.99, 1.08]	1.05*	**[1.00, 1.09]**
Mother's age								
< 25 years	Ref		Ref		Ref		Ref	
25-34 years	1.18	[0.94, 1.48]	1.22	[0.92, 1.62]	0.99	[0.95, 1.04]	1.01	[0.95, 1.06]
35+ years	1.40#	[0.95, 2.05]	1.52#	[0.96, 2.40]	1.02	[0.94, 1.11]	1.01	[0.92, 1.12]
Mother's Marital Status								
In union	Ref		Ref		Ref		Ref	
Ever in union	2.52***	[1.87, 3.40]	2.76***	**[1.97, 3.88]**	1.10*	[1.02, 1.19]	1.07	[0.98, 1.16]
Never in union	1.31	[0.93, 1.84]	1.73**	**[1.17, 2.55]**	1.16***	[1.08, 1.24]	1.23***	**[1.13, 1.34]**
Mothers ethnicity								
Kikuyu	Ref		Ref		Ref		Ref	
Luhya	1.20	[0.86, 1.69]	1.54*	**[1.06, 2.23]**	1.21***	[1.13, 1.29]	1.19***	**[1.12, 1.28]**
Luo	1.28	[0.93, 1.77]	1.83***	**[1.28, 2.62]**	1.16***	[1.09, 1.23]	1.12**	**[1.04, 1.19]**
Kamba	0.91	[0.64, 1.28]	1.16	[0.79, 1.71]	0.98	[0.92, 1.04]	1.02	[0.95, 1.09]
Other	1.49*	[1.09, 2.03]	2.08***	**[1.48, 2.92]**	1.07*	[1.00, 1.14]	1.09*	**[1.02, 1.17]**
Mother's education level								
< Secondary	Ref		Ref		Ref		Ref	
Secondary+	0.61***	[0.45, 0.81]	0.58***	**[0.43, 0.80]**	0.88***	[0.84, 0.92]	0.92**	**[0.87, 0.97]**
Mother's Parity								
One	Ref		Ref		Ref		Ref	
Two	1.41*	[1.08, 1.83]	1.23	[0.91, 1.66]	1.04	[0.99, 1.10]	1.04	[0.98, 1.10]
Three+	1.20	[0.93, 1.55]	0.91	[0.64, 1.30]	1.03	[0.99, 1.08]	1.01	[0.94, 1.07]
Pregnancy desirability								
Wanted at conception	Ref		Ref		Ref		Ref	
Wanted later	0.95	[0.76, 1.20]	0.90	[0.69, 1.17]	1.01	[0.96, 1.05]	0.93**	**[0.89, 0.98]**
Never wanted	1.45*	[1.09, 1.94]	1.08	[0.76, 1.54]	1.15***	[1.08, 1.22]	1.05	[0.98, 1.13]
Delivery place								
Health Facility	Ref		Ref		Ref		Ref	
Home	0.93	[0.73, 1.17]	0.89	[0.69, 1.15]	1.19***	[1.14, 1.25]	1.19***	**[1.14, 1.25]**
Perceived size at birth								
Normal	Ref		Ref		Ref		Ref	
Smaller	1.20	[0.91, 1.59]	0.95	[0.70, 1.30]	1.03	[0.97, 1.09]	1.03	[0.97, 1.10]
Larger	0.61**	[0.43, 0.87]	0.55**	**[0.38, 0.81]**	0.96	[0.91, 1.01]	0.97	[0.92, 1.04]
SES Category								
Poorest	Ref		Ref		Ref		Ref	
Middle	0.89	[0.70, 1.13]	0.97	[0.75, 1.25]	0.95*	[0.91, 0.99]	1.01	[0.97, 1.06]
Least Poor	1.03	[0.69, 1.54]	1.10	[0.71, 1.68]	0.90*	[0.84, 0.98]	0.96	[0.89, 1.05]
Slum								
Korogocho	Ref		Ref		Ref		Ref	
Viwandani	0.86	[0.70, 1.06]	1.20	[0.92, 1.55]	0.85***	[0.82, 0.89]	0.89***	**[0.85, 0.94]**

^1^HR = Hazard Ratio; # = p < 0.1; * p < 0.05; ** = p < 0.01; *** = p < 0.001

## Discussion

This study has documented breastfeeding and infant feeding practices in two slum settings in sub-Saharan Africa. It has also identified the factors associated with sub-optimal breastfeeding and infant feeding practices in these two slum settings. The study finds that though there is almost universal breastfeeding, exclusive breastfeeding is rare. Complementary foods are initiated too early; only two percent of children were exclusively breastfed before the age of six months and the mean age of introduction of complementary foods was one month.

In our study, we see an overall picture of universal breastfeeding with the majority of infants breastfed for at least 12 months. These findings are similar to findings in other developing countries [9,26]. However, this picture masks the unwelcome finding that the WHO recommendations with regards to breastfeeding and introduction of complementary food were largely not adhered to. Early initiation of breastfeeding following delivery, as recommended by the WHO is not universally being practiced in developing countries, despite the importance of colostrum in providing the baby with rich nutrients. The findings that close to 40% of the infants were not breastfed within one hour following delivery are in agreement with findings from studies in other developing countries including Uganda, India and Bangladesh [20,26,27]. In our setting, the main reasons stated for failure to introduce babies to breast milk immediately after birth were health-related including the mother having insufficient milk. This has also been documented in other studies [22]. Other cultural factors have been noted in other settings such as Nigeria, for example the belief that colostrum is dirty milk, hence harmful to the baby, a belief that the mother should rest and clean up first, and performance of rituals and prayers before the baby starts breastfeeding [28]. In our study though, cultural factors did not feature in the responses from the women, supposedly because our study was a quantitative, rather than qualitative study.

In this study complementary foods were initiated too early, despite two thirds of the women in our study being aware of the WHO recommended time to initiate complementary feeding. This is in line with previous studies conducted in rural Kenya, Malawi and Uganda [26,29,30], and in some other slum settings in the developing world [31,32]. In a study conducted in the late 1990s on the determinants of child nutritional status in six African countries, Madise et al. reported very low levels of exclusive breastfeeding among infants under the age of 4 months with percentages ranging from two percent in Nigeria to about 34% in Tanzania [33]. The few studies that have looked at slum settings particularly in Asia paint a similar picture [31,32]. The finding showing the persistence of early introduction of complementary feeding in the region is critical given the importance of exclusive breastfeeding to child health. Exclusive breastfeeding protects against infections such as gastrointestinal and respiratory infections, and enhances motor development in the child [4,34,35]. In a study using data from Botswana which examined the association between breastfeeding, morbidity, and malnourishment, Chikusa (1991) found that children aged 4 months or younger who had been weaned had more than 11 times the odds of having diarrhea compared with those who were still being breastfed [36]. The main reason cited for introducing complementary food early was the mother's perception of insufficient breast milk. This finding is in line with other studies from other settings which have shown that the perceived lack of sufficient breast milk is a main reason for early breastfeeding cessation or early introduction of complementary foods [31,37,38].

The variables associated with the cessation of breastfeeding during the first year of the child's life include the mother's marital status, her ethnicity, and her level of education, and perceived size at birth. The association between marital status and early cessation of breastfeeding has been reported in many studies with conflicting results [22,23]. In this study, women who were not in union, particularly those who were formerly married were more likely to stop breastfeeding their infants than women who were in union. It has been suggested that the association between marital status and breastfeeding cessation may be due to the presence or absence of social, emotional and economic support of a partner [39]; however, these factors were not assessed in our study. A more plausible reason in Kenya, where HIV is high, is that a disproportionately large number of formerly married women are HIV positive and many women in this situation were until recently advised to exclusively breastfeed their infant for 6 months and then to rapidly wean [40].

The evidence of the association between a mother's level of education and the duration of breastfeeding also varies [20,24,25]. In this study, lower than secondary level education was associated with earlier cessation of breastfeeding. While it is not very clear why this is the case, higher education may be associated with higher knowledge and practice of positive health behaviour. Higher HIV prevalence among those with less than secondary level education, especially those with no education at all in our setting [41] may be associated with early cessation of breastfeeding. We also observed an association between ethnicity and breastfeeding cessation. All other ethnic groups apart from the Kamba were more likely to stop breastfeeding their infants compared to Kikuyu women. There is no established reason for this but it could be multi-factorial, including cultural practices related to breastfeeding and child rearing. Further, HIV prevalence, which may affect breastfeeding practices, has also been documented as higher amongst the Luo and Luhya ethnic groups in this slum setting compared to the Kikuyu ethnic group [41]. Additionally, evidence from the study areas indicates that Kikuyus have lower fertility compared to other ethnic groups. Prolonged breastfeeding may explain or be explained by lower fertility. Mothers who get pregnant while breastfeeding are more likely to stop but, also mothers who breastfeed for longer period have lower chances of getting pregnant [42]. Additionally, better child health outcomes have also been documented among the Kikuyu's compared to other ethnic groups in this study setting [14] and our findings may indicate that the Kikuyus have better health-related behaviours and practices than most of the other ethnic groups in the study area. The association between birth size and the duration of breastfeeding has not been studied in depth. Our study found that children who were perceived to be larger at birth were less likely to be stopped from breastfeeding earlier. This is similar to a US study, infants who were breast-fed for less than 4 months were smaller at birth than those who were breast-fed for 4 months or more [43]. The factors behind this association in our study setting need further investigation.

Predictors of early introduction of complementary foods include the child's sex; the mother's marital status, her ethnicity, and her level of education; the desirability of the pregnancy of the index child, the place of delivery and the slum setting. Boys were more likely to be introduced to complementary feeding early compared with girls. Anecdotal evidence indicates that boys are introduced to complementary foods early because breast milk alone does not meet their feeding demands. Having never been in union/married was associated with higher risk of early introduction of complementary foods. A positive association between being married and exclusive breastfeeding has been documented in other studies [44]. As in the case of the duration of breastfeeding, this may be associated with social, emotional and economic support of a partner [39]. Similar to the finding related to the duration of breastfeeding, all other ethnic groups apart from the Kamba group, were more likely to initiate complementary foods earlier than the Kikuyus. This may be related to cultural practices and other factors such as HIV status as described for duration of breastfeeding. While a few studies have linked the mother's education with early introduction of complementary foods [27], similar to our study, the negative influence of a mother's low education on early introduction of complementary foods has been observed in many studies in other settings, suggesting a need for education and health promotion to change these harmful feeding practices [21,45,46].

Slum of residence, was associated with the timing of introduction of complementary foods. Mothers from Viwandani, were at lower risk of introducing complementary foods before six months. This may be because Viwandani, being in the industrial area and attracting labourers to the industries is likely to have more educated people (other than the mother) for example the father and other household members, who may affect infant feeding practices. Mothers who delivered at home were more likely to introduce complementary foods earlier than those who delivered in a health facility. Mothers who deliver in a health facility in most cases receive breastfeeding counselling, especially with the revitalisation of the Baby Friendly Hospital Initiative (BFHI) from 2007 aimed at promoting optimal breastfeeding practices. The BFHI has been found to be effective in several settings in the developing world [47]. BFHI, is being revitalized in Kenya in the National Strategy on Infant and Young Child feeding [12], and it may be playing a role in encouraging mothers to exclusive breastfeeding their infants in the first 6 months of life. Since the BFHI initiative was introduced, there has been potential improvement in the proportion of children exclusively breastfed from 13% in 2003 to 32% in 2008 [10]. The positive association between pregnancy desirability and complementary feeding has barely been previously studied. In our study, infants who were unwanted at conception but were wanted later were less likely to be introduced to complementary foods early. The association between pregnancy desirability and breastfeeding and complementary feeding practices needs further investigation.

Urban slum settings present unique challenges with regards to breastfeeding and infant and young child feeding practices due to their physical and socio-economic characteristics. In these informal settings, basic government services including health care services are limited and this, coupled with financial constraints, leads to a substantial proportion of women in these slums giving birth at home or at informal private health facilities [48,49]. This means that most of these slum women are systematically excluded from government initiatives such as those aimed at promoting optimal breastfeeding and infant feeding practices, based at health facilities such as the BFHI mentioned above, which involves counselling of mothers on infant and young child feeding around the time of delivery. Another unique characteristic of slum settings is limited livelihood opportunities [15] hence food insecurity. As indicated in this study and in other slums such as in India [31], one key reason for initiating complementary foods too early is due to the mother having inadequate breast milk. While the important role of hormones and the psychosocial status of the mother in lactation is well established, though limited evidence exists, volume of milk produced may also be related to maternal nourishment. A review of breastmilk volumes and composition among poorly nourished communities indicated that milk volumes were lowest in communities with poor levels of nutrition and poor living conditions [50].

Potential interventions to address the unique challenges in the slum settings should address both access issues and the socio-economic limitations. A potential intervention to counteract the systematic exclusion from basic government services may include, home-based counselling of mothers on infant and young child feeding by community based health workers and/or supporting the (informal) private service providers for instance through training programs to offer services according to established government guidelines such as those on breastfeeding. The effectiveness of such interventions in health care delivery, including promotion of optimal infant feeding practices in resource-constrained settings has been indicated [51]. To enhance adequacy of milk produced by the mothers, potential interventions may be to enhance maternal nourishment through ensuring food security. This may be through appropriate income generating activities to enhance livelihoods. Food supplementation has also been found to enhance breastmilk volume [50]. Additionally, interventions that empower the new mother by demonstrating correct breastfeeding techniques, ways of stimulating breast milk production, and counselling on proper nutrition may improve breastfeeding practices [52].

Limitations in this study relate to missing values in some of the variables particularly the socio-economic status variables. Appropriate measures were taken in the analysis to minimize bias as indicated above. It would have been important to follow-up the children for a longer period to establish complete duration of breastfeeding in line with the WHO recommendation that breastfeeding should continue for two years or beyond. This was however not done and children were only followed up till they were slightly more than one year old. Despite these limitations, this study has key strengths that are worth mentioning. The study provides important information on infant breastfeeding and feeding practices in informal settings in sub-Saharan Africa, for which there is a dearth. A key strength of this study lies in its longitudinal nature, minimising recall bias that may be associated with cross-sectional studies. The study involved rigorous follow-up hence information for most of the children was updated by the end of the follow-up period, reducing bias due to loss to follow-up. The study involved a census of all children born to mothers in two defined geographical areas; hence there was minimal bias due to sampling error.

## Conclusion

This study presents important findings on breastfeeding and infant feeding practices and determinants of sub-optimal practices in informal settings in sub-Saharan Africa. This is timely and critical as such evidence is currently rare. The study finds that despite universal breastfeeding in this population, WHO breastfeeding and infant feeding recommendations are rarely adhered to. It is important therefore to develop interventions targeting women, health care workers and policy makers aimed at bridging the gap between current breastfeeding and infant feeding practices in the informal settings and WHO recommendations. It is evident from this study that breastfeeding and infant feeding patterns are associated with child, maternal and household level factors and it is crucial to understand and reduce the inequalities. Interventions and further research should address inequalities including gender, ethnicity, access to and utilization of health care facilities, socio-economic status and family planning. This study only looked at early introduction of complementary food; further investigation on late introduction of the same is needed.

## List of abbreviations

**APHRC: **African Population and Health Research Center; **BFHI: **Baby Friendly Hospital Initiative; **HIV: **Human Immuno-deficiency Virus; **NUHDSS: **Nairobi Urban Health and Demographic Surveillance System; **HR: **Hazard Ratio; **TBA: **Traditional Birth Attendant; **UNICEF: **United Nations Children's Education Fund; US: United States; **WHO: **World Health Organization.

## Competing interests

The authors declare that they have no competing interests.

## Authors' contributions

EWK-M:Design of the study, project management, data analysis, writing of the manuscript and approval for submission; NJM: Principal Investigator of the project, design of the study, analytic guidance, reviewing of the manuscript and approval for submission; J-CF: Design of the study, overall project co-ordination, reviewing of the manuscript and approval for submission; CK: Design of the study, project management, review of the manuscript and approval for submission; MK: Data management and analysis, review of the manuscript and approval for submission; TG: Writing of the manuscript and approval for submission; NY: Writing of the manuscript and approval for submission. All authors read and approved the final manuscript.

## Pre-publication history

The pre-publication history for this paper can be accessed here:

http://www.biomedcentral.com/1471-2458/11/396/prepub
